# Crawling at High Speeds: Steady Level Locomotion in the Spider *Cupiennius salei*—Global Kinematics and Implications for Centre of Mass Dynamics

**DOI:** 10.1371/journal.pone.0065788

**Published:** 2013-06-21

**Authors:** Tom Weihmann

**Affiliations:** Institute of Sports Science, Science of Motion, Friedrich-Schiller University, Jena, Germany; University of Zurich, Switzerland

## Abstract

Spiders are an old yet very successful predatory group of arthropods. Their locomotor system differs from those of most other arthropods by the lack of extensor muscles in two major leg joints. Though specific functional characteristics can be expected regarding the locomotion dynamics of spiders, this aspect of movement physiology has been only scarcely examined so far.

This study presents extensive analyses of a large dataset concerning global kinematics and the implications for dynamics of adult female specimens of the large Central American spider *Cupiennius salei* (Keyserling). The experiments covered the entire speed-range of straight runs at constant speeds. The analyses revealed specific characteristics of velocity dependent changes in the movements of the individual legs, as well as in the translational and rotational degrees of freedom of both the centre of mass and the body.

In contrast to many other fast moving arthropods, *C. salei* avoid vertical fluctuations of their centre of mass during fast locomotion. Accordingly, aerial phases were not observed here. This behaviour is most likely a consequence of optimising energy expenditure with regard to the specific requirements of spiders' leg anatomy. A strong synchronisation of two alternating sets of legs appears to play only a minor role in the locomotion of large spiders. Reduced frequency and low centre of mass amplitudes as well as low angular changes of the body axes, in turn, seems to be the result of relatively low leg coordination.

## Introduction

Spiders, in particular the Araneomorphae suborder, are next to insects one of the largest terrestrial groups within the arthropod phylum. Within this phylum they are a major predator class. However, in contrast to insects, knowledge about the locomotion of spiders on firm substrates is still rudimentary. In general, spiders are envisioned to build webs, which is widely considered determining of their lifestyle and locomotion. However, there are many species within a couple of families that do not build webs at all. They ambush their prey and attack it suddenly by a fast sprint or a directed jump, without exhibiting obvious specific adaptations of their locomotor system to this life style. In contrast, web building spiders show strong adaptations of their locomotor system to their specific substrates [1], although they are in general poorly prepared for faster locomotion on firm horizontal substrates [2]. Furthermore, almost all non-web building spider species are sit and wait predators. That is why for vagrant spiders the ability for fast terrestrial locomotion is particularly important, since they need to be prepared for both sudden attacks and escapes. In these situations, spiders reach maximum speeds, which are comparable to those of the fastest insects, although these insect species like cockroaches and ants are particularly adapted to fast running requirements [3]–[6].

### Hydraulics

A specific characteristic of spiders is their lack of extensor muscles in two major leg joints, namely the femur-patella-joint and the tibia-metatarsus-joint. In unloaded legs these joints can be extended only by means of hydraulic pressure, which is generated in the prosoma and transmitted via lacunae to the respective joints. Although there are alternative extension mechanisms in legs with ground contact [7], hydraulic pressure also seems to be important for leg movement during stance (cp. [7] for a review). Besides some related groups [8] spiders are the only animals that combine hydraulics and an exoskeleton consisting of stiff segments. From a technical point of view, hydraulics is very interesting and finds a wide range of applications right down to the microscopic scale. In the course of elaborating miniaturisation of mechanical principles, biomimetic approaches referring to the joint design of spiders has come into the focus of engineers' attention [9]–[11]. Therefore, it seems peculiar that fast spider locomotion and spider locomotion in general, has hitherto been examined so little.

### Leg coordination

Ward and Humphreys showed in 1981 that differently adapted spider species move their legs in different coordination patterns [12]. The vagrant species *Trochosa ruricolla* runs with two alternating sets of four diagonally adjacent legs, which is quite similar to fast running insects, whereas *Lycosa tarantula*, a markedly burrow dwelling species, showed distinct deviations from this pattern. During fast running the latter preferred the metachronal wave mode of leg coordination, which is characterised by intermediate phase relations between adjacent ipsilateral legs. Similar patterns were also identified by Moffet and Doell [13] for *Pardosa tristis* running at medium speeds and by Shultz [14] for the terrestrial locomotion of *Dolomedes triton* and *Lycosa rabida*. Spagna et al. [15] identified for the two small spider species *Hololena adnexa* and *Hololena curta*, which seemingly use approximately alternating gait patterns, impressive running speeds up to 70 body-lengths per second. At top speed these spiders occasionally show aerial phases. Although larger species are also perfectly capable of jumping distances of several body lengths [16], until now it has remained uncertain whether large species also show aerial phases during running.

### Model-based frequency relations

If we look at the established models for legged locomotion, both sagittal and horizontal spring-mass models [17]–[19], as well as the inverted pendulum model [20] require rhythmic oscillations of the centre of mass (COM). Though the variability of these oscillations' frequencies strongly depends on leg coordination, spring-mass and inverted-pendulum dynamics always imply distinct frequency relations between the fluctuations of the body's translational and rotational degrees of freedom (DOF). These frequency relations were particularly well demonstrated for cockroaches, which adopt spring-mass dynamics at medium and high speeds. Here, the frequencies of lateral fluctuations, yaw and roll scale with the stride frequency of the walking legs. In contrast, that of anterior-posterior and vertical fluctuations, as well as pitch, adopt values double the stride frequency [21]–[24]. In the vertical direction, distinct coupling of kinetic and potential energy is a consequence (out of phase in the inverted pendulum and in phase in the spring-mass model). Beyond the assumed conservation of energy, sagittal and horizontal spring mass models provide self stability as a further advantage [19], [25]. This allows negotiation of small surface irregularities, obstacles, or other small disturbances without any neuronal feedback.

### Leg characteristics

**Figure 1:**
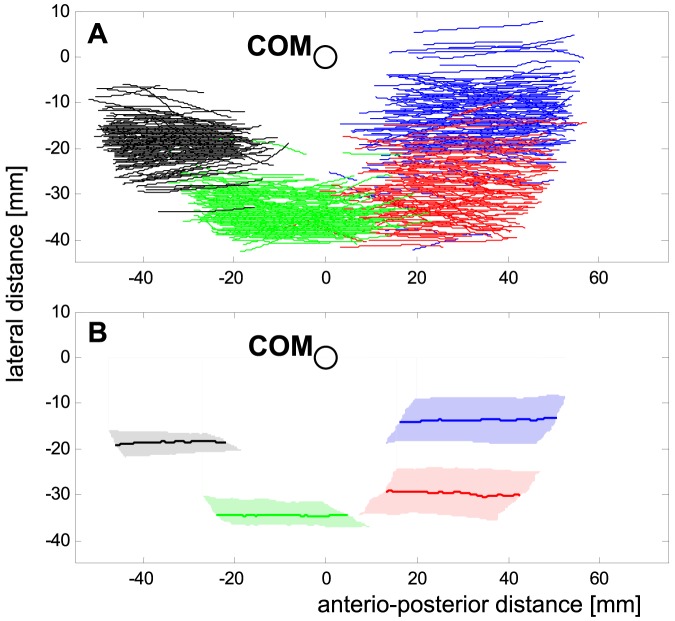
Movements of the legs' tarsi relative to the centre of mass (COM) seen from above. **a**) Measured trajectories, **b**) average contact areas with median trajectories of the legs' tarsi with respect to the COM, i.e., regions with respect to the COM where ground contact primarily occurred. Front legs are depicted in blue, second legs in red, third legs in green and hind legs in black. The light-coloured areas in b are bounded in the anterior-posterior direction by the medians of the anterior and posterior extreme positions for a given lateral distance to the COM. In the lateral direction, the boundaries are the 25% and the 75% quartiles of the lateral distributions for distinct anterior-posterior positions of the tarsi.

Most spider species have comparatively long legs. The legs are arranged roughly radially symmetric around the prosoma with the frontal leg pairs pointing anteriorly and the hind legs posteriorly. Only the 3^rd^ legs (from the anterior) are held mainly laterally (cp. Figure 1). Anatomically each leg consists of 7 segments. However, during locomotion, the proximal segments coxa and trochanter form the functional hip, while the patella and tibia act predominantly as one segment and the tarsus acts primarily as an attachment device. Thus, from a biomechanical point of view, spider legs can be reduced to a functionally three-segmented bow-shaped structure. As a result of this structure, spider legs are more compliant (see discussion) than similarly sized zigzag shaped or two-segmented legs. Such configurations, in turn, are the structural bases of many insect legs. Furthermore, no elastic structures could be identified in spider legs that would be potentially suitable to store and release locomotion energy [8]. These characteristics do not support the anticipation of spring-mass like dynamics for spiders.

### Hypotheses

In this study, terrestrial spider locomotion shall be examined with emphasis on the impact of the specific spider physiology on global kinematics for the entire speed-range. For the first time, all translational and rotational DOFs of the COM and the body axes of a fast running spider species, the well-known Central-American species *Cupiennius salei*, are examined. The results allow for comparisons with predictions made by the aforementioned models. If the DOF's frequency relations come close to that predicted by spring-mass models or the inverted pendulum model, then these models could be considered to sufficiently describe the dynamics of spiders. In contrast, if the fluctuation frequencies measured in this study differ significantly from those expected from model predictions, then spider dynamics must differ from those of cockroaches and other animals with locomotion dynamics matching these models' dynamics.

The approach presented here will help to answer two questions. First, is there an established locomotion model that fits best with real spider motions? This would imply a strong coupling between the fluctuation frequencies of the DOFs at all running speeds. If there is no distinct coupling then the second question is: Are there alternative locomotion dynamics that might have positive effects with respect to the very sprawled leg posture of spiders or due to lacking elastic elements?

Body dynamics, and consequently its kinematics, are the result of all external forces acting on the animals. It is largely determined by the activities of the walking legs. Since body dynamics as well as leg coordination usually vary with speed [26], [27], a complete analysis of animal locomotion will also require determining the mean leg coordination of the single leg pairs with respect to the COM, the speed dependent changes of the stride frequencies, as well as those of the contact and swing durations. Lastly, spiders are known for particularly low and variable temporal leg coordination [2], [28]–[30], thus the phase relations between the different legs also had to be determined.

The present study provides a basis that allows for determining potential nonlinear dependencies of several parameters with respect to running speed. Here the advantages of 2D and 3D kinematic analyses were combined, as 2D analyses allow for larger sample sizes with relatively lower digitisation effort, while only 3D analyses permit the evaluation of all kinematic fluctuations of the COM. With both approaches it was possible to investigate leg and body kinematics of *C. salei* during provoked escape runs by using high-speed video and motion-analysis techniques. The results, in turn, allow drawing inferences with respect to underlying dynamic principles.

## Materials and Methods

### Animals

Eight adult female specimens of the Central American hunting spider *Cupiennius salei* were used in the experiments. They were bred in the laboratories of F. G. Barth (Vienna). *C. salei* usually populates tropical forest plants with large leaves [31], [32], but they are also often found on the forest floor, particularly in sub-adult stages. As they are not known as outstanding runners and quickly become exhausted, the specimens had to be exchanged regularly after a couple of trials. Nevertheless adult females achieve considerable velocities with maximum values of up to 0.7 m/s [33]. Furthermore, *C. salei* is one of the best-examined spider species. Several work groups examined aspects of sensory physiology, neurobiology, developmental issues, behaviour, orientation, muscle physiology and, to some extent, even locomotion [16], [32], [34]–[38]. *C. salei* is a species of the family Ctenidae, nevertheless it is characterised by its long frontal leg pairs, similar to huntsmen spiders, and both sexes show a distinct dimorphism as is typical for most spider species. Female specimens were chosen since most former studies regarding spider locomotion also only focussed on adult females. All spiders were of similar size (3.24 cm±0.29 cm) and mass (3.56 g±0.63 g). The spiders were kept at room temperature (21° to 23°C) and fed on crickets of appropriate size. Water was given *ad libitum*.

### Specifications of measured quantities

**Figure 2:**
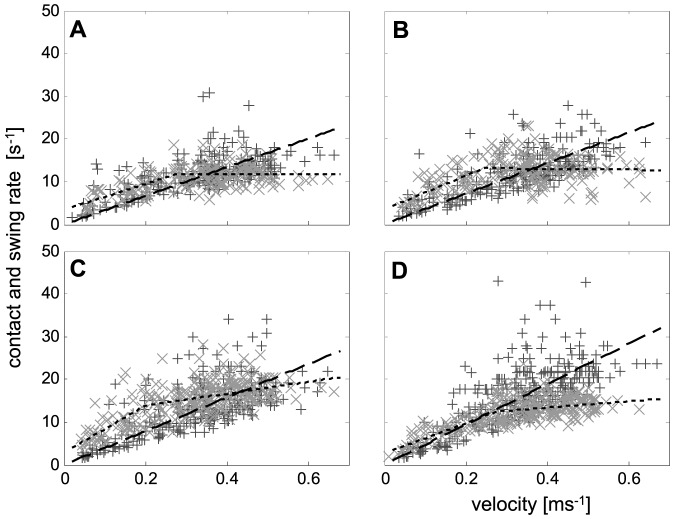
Velocity dependencies of the inverted contact durations (*t_C_* ^−1^, black ‘+’ symbols) and the inverted swing durations (*t_S_* ^−1^, grey ‘×’ symbols). **a**) Front legs, **b**) second legs, **c**) third legs, and **d**) hind legs. Dotted regression lines represent the velocity dependencies of the inverted swing durations and dashed regression lines represent that of the inverted contact durations.

All measures, which will be used in the following for the characterisation of spiders' kinematics and dynamics, shall be defined here. Stride length (*s_T_*) is the distance from take-off to touch-down of a certain legs tarsus, it is equivalent to the distance travelled by the COM during a contact and a subsequent swing phase or vice versa. Contact length (*s_C_*) is the distance traversed by the COM during the contact phase of a certain leg and swing length (*s_S_*) is the distance the COM travels during swing phase. All these lengths relate to temporal measures. The most basic temporal measures, contact duration (*t_C_*) and swing duration (*t_S_*), change highly non linear with speed. In order to linearize them, regressions were made on the basis of the inverses (*t_C_* ^−1^ and *t_S_* ^−1^). The inverted contact duration (*t_C_* ^−1^) increases linearly with running speed. As contact duration tends towards infinity with decreasingly slower speeds, the zero-crossing of its inverse must lie in the point of origin. The inverted swing durations showed relatively high slopes at lower speeds and low slopes at high speeds. Both speed dependencies were adopted as linear (Figure 2). The point of intersection indicates a transitional velocity (cp. results and discussion).

**Figure 3:**
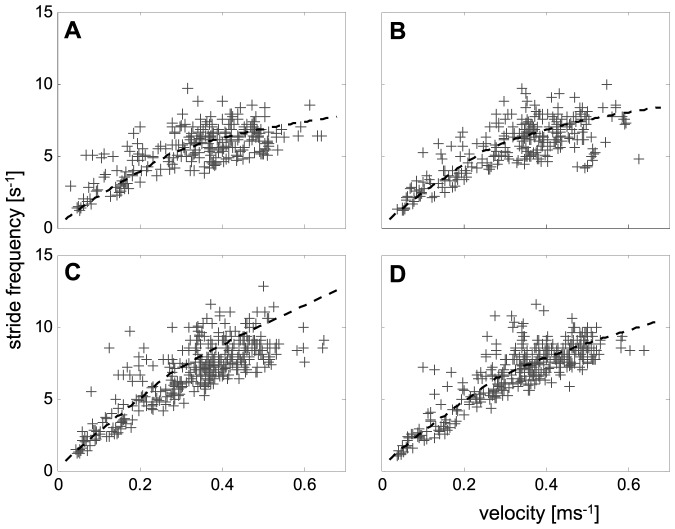
Velocity dependencies of the stride frequencies (*f_T_*) of all leg pairs. **a**) Front legs, **b**) second legs, **c**) third legs, and **d**) hind legs. The approximations (dashed lines) were calculated from the linearized regressions of *t_C_* ^−1^ and *t_S_* ^−1^ (cp. Figure 2 and Materials and Methods section).

**Figure 4:**
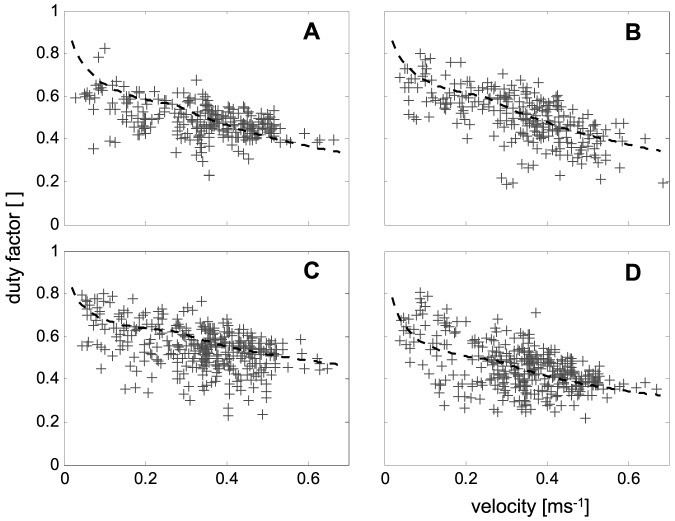
Velocity dependencies of the duty factors (*Du*) of all leg pairs. **a**) Front legs, **b**) second legs, **c**) third legs, and **d**) hind legs. The approximations (dashed lines) were calculated from the inverted regressions of *t_Ci_* ^−1^ and *f_Ti_* (cp. Figures 2, 3 and Materials and Methods section), i.e. *t_Ci_* and *t_Ti_* of the single legs.

Stride frequencies and duty factors derive from these durations and rates, respectively. Their velocity dependencies are highly non-linear as well. The stride frequencies (*f_Ti_*) can be calculated as *f_Ti_* = *t_Ti_* ^−1^ = (*t_Ci_*+*t_Si_*)^−1^ with the index *i* indicating the respective leg number. Their slopes are linear and relatively steep at low speeds and decrease in a non-linear manner at speeds above the transition point (Figure 3). With *t_Ti_* being the stride duration, i.e. the inverted stride frequency of the legs, duty factors were calculated conventionally (*Du_i_* = *t_Ci_*/*t_Ti_*) for all leg pairs. Due to the specific characteristics of *t_Ci_* and *t_Ti_*, their velocity dependencies each displayed graphs consisting of two attached hyperbola. At low velocities, the curvatures of the graphs were higher than above the transition speed (Figure 4).

### Experimental setup

For the experiments, we used two different approaches for the acquisition of 2D and 3D kinematics. Twenty sequences were recorded in 3D and a further 60 sequences in 2D. In total, 146 steps of the frontal legs, 139 steps of the 2^nd^, 204 steps of the 3^rd^ and 210 steps of the hind legs could be analysed. Post-triggered high-speed video cameras of the type HCC-1000 (VDS-Vosskühler GmbH, Osnabrück, Germany) with a resolution of 1024×512 pixels each, were utilised at a sample rate of 308 frames per second for both setups. During 2D recording one single camera was mounted above the experimental area viewing perpendicular onto the substrate. During 3D recordings, five cameras were used simultaneously. Three cameras were all positioned around the sample area at a height of about 40 cm above the surface. Two cameras were positioned about 1 m above the sample area to provide top views. For each sequence these two perspectives were chosen for digitisation, which provided the best views of the markers (see below) and the tarsi. In most cases this was one of the top and one of the side views. Spiders running on a narrow track bordered by barriers always strived to scale these barriers when coming close to them. Even if they could not attach to the walls, the spiders' gait patterns were considerably distorted. Spiders running on elevated running tracks strived to come off whenever they reached the rim. Hence, in this study, the spiders were allowed to move freely in an experimental arena. However, introduced in this artificial and unfamiliar setting, the spiders mostly tried to hide themselves by pressing their body to the ground and had to be spurred on to run (see below). The arena was about 120 cm long and 60 cm wide. It was covered by fine sandpaper providing sufficient skid resistance and surrounded by an 8 cm high barrier, which was made of balsa wood. The sample area was illuminated by two 500 W spotlights allowing for short exposure time and clear single frame greyscale images. By making use of close-up lenses (Componon-S 2.8/50; Jos. Schneider Optische Werke GmbH, Bad Kreuznach, Germany), the cameras covered an area of about 28 cm×13 cm in 2D and 25 cm×15 cm in 3D, so tracking accuracy was about 0.3 mm. An accuracy of at least 0.15 mm can be assumed since the used digitisation software is able to compute sub-pixel resolutions via interpolating algorithms [39], [40]. Depending on running speed and direction, sequences of 2–8 strides could be recorded. To calibrate the video sequences, calibration bodies made of LEGO™ (Billund, Denmark) blocks were used. These blocks have constant dimensions of 1/10^−3^ inch precision. In 2D recordings the dimensions of the calibration body were 160 mm×32 mm, and in 3D recordings the dimensions were 128 mm×64 mm×27 mm. The calibration bodies provided frameworks of coplanar (2D) or non-coplanar (3D) points that allowed for the calibration of the sequences with a mean divergence of lower than 0.5 pixels.

**Figure 5:**
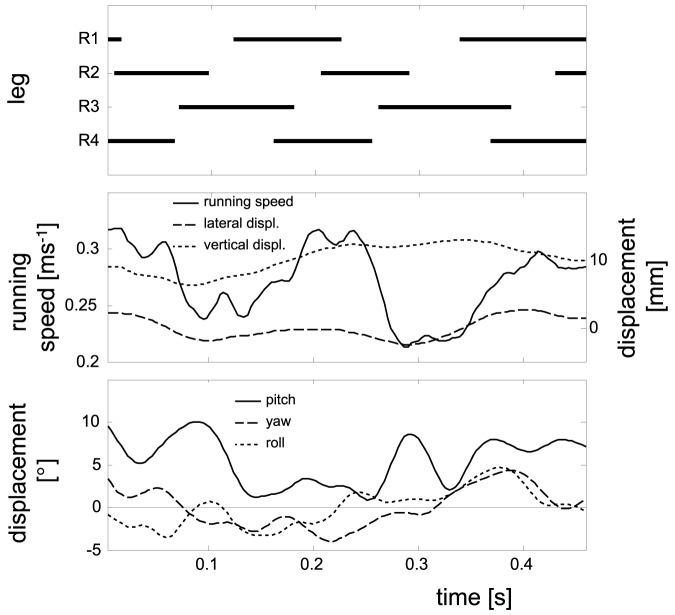
Sample trajectory of changes in the translational and rotational DOFs in a female specimen of *C.* *salei* running at an approximate net speed of 0.28 ms^−1^. The upper graph shows the stepping pattern of the right legs. It is roughly consistent with a metachronal wave model (*f_T1_*: 4.7 s^−1^; *f_T2_*: 4.9 s^−1^; *f_T3_*: 5.0 s^−1^; *f_T4_*: 5.0 s^−1^). The middle graph shows fluctuations in forward velocity (left y-axis, solid line; approx. 4.4 s^−1^), dorso-ventral (dotted line; approx. 2.2 s^−1^) and lateral (dashed line; approx. 4.4 s^−1^) excursions of the COM (right y-axis). Thereby, lateral excursions to the left are indicated by positive values. The lower graph shows the temporal changes of the rotational DOFs (pitch: solid line; yaw: dashed line; roll: dotted line). Here, the lower frequencies are partially superimposed by frequencies with higher fluctuation frequencies (pitch: approx. 2.2 s^−1^ superimposed by 10.9 s^−1^; yaw: approx. 2.2 s^−1^; roll: approx. 2.2 s^−1^ superimposed by 6.5 s^−1^).

Three hemispheric reflecting marker points with a diameter of 2 mm each were glued onto the prosoma. One of them was placed centred on the anterior rim just behind the posterior-median eyes. The other two markers were placed right and left onto the posterior rim of the prosoma. As the COM is located near the hind leg's coxae [30], [41], its approximate position seen from above is halfway between the posterior marker points. These three marker points allowed for the acquisition of both the translational fluctuations of the COM in all spatial dimensions and the rotational DOFs of the body. All recorded running sequences were provoked escape runs. The escape responses were caused by waving hands, blowing air puffs or by slight touches of a soft brush onto the opisthosoma. To evoke forward directed escapes, all disturbances were exerted towards the posterior end of the spiders. Only those intervals of constant speed runs with deviations less than 15° from a straight line were analysed. Under arena conditions the spiders started mostly from a resting position and they had to slow down at least when the surrounding barrier was reached. Nevertheless, the animals gained almost constant running speeds just after one or two strides and often maintained this speed for a couple of strides. Only such phases without significant accelerations or decelerations were analysed. Intervals at which the mean running speed changed by more than 15% between adjacent strides were excluded from analyses (cp. Figure 5).

### Video analysis

We digitised the body markers and additionally the distal tips of all tarsi during ground contact by using WINAnalyze 2.1 (mikromak®, Berlin, Germany) software. The right-handed system of coordinates was defined with the z-axis being parallel to gravity, the x-axis perpendicular to the z-axis in the direction of motion and the y-axis being perpendicular to the xz-plane. Since the animals fled in any direction, all digitised positional data was rotated in this coordinate system around the z-axis. In order to analyse the movements of the legs relative to the COM, the left and right legs were not discriminated, thus, lateral kinematics of left legs were mirrored. Velocities, accelerations and angles were calculated with MATLAB 7.2 (MathWorks™, Massachusetts, USA). The COM of large spiders lies centrally at the posterior rim of the prosoma [30], [41]. Here its position is assumed as lying within the hind end of the prosoma 2 mm below the upper surface halfway between the posterior body markers. Running speed was calculated by using the derivative of the COM's positional data with respect to time. This was determined by smoothing primary data by fitting gliding second order polynomials to the time series, including four adjacent points on both sides at each instance in time. The longitudinal body axis was established by connecting a line from the anterior body marker to the point halfway between the posterior body markers. The contact phase of a certain leg was defined as the interval where the tarsus had ground contact and was not sliding across the substrate. Dragging of the rear feet could be regularly observed though the actual contact phase had already ended. These dragging periods were assigned to the swing phase. Fluctuation frequencies of the COM (anterior-posterior, laterally and vertically), of the body axis (yaw and pitch), and of the transverse axis (roll), i.e. the line between the posterior body markers, were determined by analysing the spectrum calculated with an FFT-algorithm. Prior to these analyses, all data were high-pass filtered by using a Butterworth filter with a cut-off frequency of 0.5 s^−1^ to eliminate offset. Due to multiple superimposed oscillations (cp. Figure 5), the spectra often showed two or three maxima. In such cases the frequency with the highest energy content, i.e. the highest peak, was considered as representative. In the direction of motion, the horizontal position as a function of time is dominated by the average forward speed rather than fluctuations about it. Therefore, the frequency of anterior-posterior fluctuations was acquired by analysing the time series of the running velocity, i.e. the first derivative with respect to time. All other frequencies were achieved by analysing the time series of coordinates or those of angles to the substrate. Amplitudes were determined by measuring the differences between adjacent local minima and maxima. If more than one of such deflections could be determined during one sequence, then their mean value was calculated.

### Statistics

All statistics were calculated with embedded MATLAB functions (MATLAB 7.2; MathWorks™, Massachusetts, USA) or self developed scripts. For evaluation of the data, normal descriptive as well as circular statistics were applied. Translational and positional data were tested to determine whether or not each parameter matched a normal or at least a symmetric distribution. If so, as a measure for the central tendency, the mean with standard deviation is given; if not or if the sample size was low, the median as well as the lower and upper quartile is presented. Similarly, for multivariate comparisons one-way ANOVA with adjacent Tukey-Kramer post-hoc or a nonparametric one-way ANOVA after Kruskal and Wallis with adjacent comparison of average group ranks was applied.

Linear and bi-linear least squares fitting were applied for the description of velocity dependent changes of inverted contact durations (*t_C_* ^−1^; see results section) and swing durations (*t_S_* ^−1^) by using MATLABs curve fitting toolbox (cftool.m). For bi-linear fitting, different linear regression lines were fitted for velocities below and above a transitional speed (*v_×_*), while the transitional speed was defined as the position of the intersection point of the lines along the abscissa. In the course of fitting, the position of the intersection point was not fixed and resulted from the best overall fit of the bi-linear outcome (cp. Figure 2). Eventually *v_×_* was calculated by the quotient 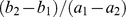, with *a_1_* being the slope and *b_1_* the y-intercept of the regression line at speeds below and *a_2_* and *b_2_* at speeds above the transition.

For comparisons of slopes and intercepts of different regression lines or to test these measures against the null hypothesis respectively, the procedures described in Sachs [42] were used. These methods rely on the calculation of values of a *t*-distribution by determining the quotient of the difference between, both regressions and their standard error, plus subsequent comparison of the calculated values with critical limits. Unless specified, all tests were done at the 5% confidence limit.

Since one focus of this study lies on stride sequences of periodical gaits, the temporal relations of the single legs' motions were described by phase relations *θ_i_*. Touch-down was chosen as critical, i.e. the occurrence of this distinct event of one leg within the stride period of another leg was examined. A circular distribution results when gathering many single events. This distribution lies on the circumference of a circle of unit radius. Following Fisher [43], the circular means of such distributions can be calculated as 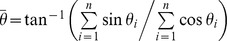. A value quantifying the strength of the coupling between the respective legs is the mean resultant length given by 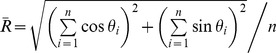. The circular standard deviation is a value of variability equivalent to the linear standard deviation [43], which can be calculated as 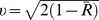. Furthermore, the confidence interval was calculated at the 95% level. Circular means, circular standard deviations, and confidence intervals calculated in radians are presented as fractions of the stride period.

## Results

The specimens reached constant running velocities of up to 0.6 ms^−1^, even if fast running sequences lasted mostly only a couple of strides. During these velocity plateaus, median running speeds attained about 0.32 ms^−1^ and the inter-quartile range ranged from 0.2 ms^−1^ to 0.4 ms^−1^. The mean height of the COM did not change with running speed; its median value was about 10.1 mm (quartiles: 8.7 mm; 11.4 mm) above the substrate over a stride.

### Spatio-temporal measures

During stance, both the anterior-posterior and the lateral positions of the tarsi differ significantly between the leg pairs (Figure 1a). Applying Levene's test, the lateral variability displays significant differences between the frontal and the rear leg pairs. Thus, the variances of leg pairs 1 and 2 were significantly larger than that of the 3^rd^ and 4^th^ legs. Many of the examined measures, such as stride duration, the inverted swing duration, and the duty factor showed clear nonlinear velocity dependent changes. As mentioned in the Materials and Methods section, linearization of at least the fundamental measures were attempted. By using the linear (*t_C_* ^−1^) and bilinear (*t_S_* ^−1^) velocity dependencies of the inverted contact and swing durations it was possible to calculate the non-linear dependencies of the stride frequencies (*f_Ti_*) and that of the duty factors (*Du_i_*) of the single legs (cp. Figures 3, 4 and see Materials and Methods section). Since slopes of the inverted swing durations of the 1^st^ and 2^nd^ legs at higher speeds were approximately zero and those of the 3^rd^ and 4^th^ legs were significantly larger (Figure 2; Table 1), the velocity dependencies of the stride frequencies also differed. Thus, the stride frequencies of the hind legs show nearly linear slopes throughout the entire speed-range, while those of the frontal leg pairs are clearly non-linear and increase slower at higher running speeds (cp. Table 2).

**Table 1:** Parameters (slope a, and y-intercept b) for linear regressions of kinematic measures.

parameter		1st legs	sig	2nd legs	sig	3rd legs	sig	4th legs	sig
*t_C_* ^−1^ [s^−1^]	a	33.47 (32.01, 34.93)	34	35.72 (34.31, 37.12)	4	39.29 (37.62, 40.97)	14	47.16 (45.67, 48.65)	123
	b	-		-		-		-	
*t_S_* ^−1^ [s^−1^] at *v*<*v_×_*	a	29.95 (20.18, 39.73)	3	39.32 (24.31, 54.34)	3	55.44 (36.54, 74.35)	124	35.5 (28.7, 42.3)	3
	b	3.45 (1.76, 5.14)	-	3.521 (1.184, 5.859)	-	2.786 (0.3709, 5.2)	-	2.641 (1.429, 3.852)	-
*t_S_* ^−1^ [s^−1^] at *v*>*v_×_*	a	−0.16 (−5.37, 5.05)	34	−1.0 (−7.25, 5.25)	34	13.86 (8.72, 19.0)	12	7.34 (3.04, 11.64)	12
	b	11.73 (9.57, 13.89)	-	13.42 (10.86, 15.98)	4	11.1 (9.09, 13.11)	-	10.48 (8.72, 12.25)	2
*s_S_* [mm]	a	62.6 (49.18, 76.02)	3	76.61 (51.16, 102.1)	34	38.67 (31.26, 46.09)	124	51.29 (43.62, 58.96)	3
	b	12.89 (7.33, 18.45)	-	7.33 (−3.55, 18.22)	-	8.72 (5.76, 11.69)	-	8.72 (5.62, 11.82)	-
*s_T_* [mm] at *v*>*v_×_*	a	141.5 (108.7, 174.3)	34	110.8 (75.25, 146.3)	-	89.06 (59.37, 118.8)	1	78.11 (52.3, 103.9)	1
	b	10.31 (−4.05, 24.67)	-	19.5 (4.17, 34.83)	-	14.88 (0.94, 28.82)	-	20.39 (8.3, 32.49)	-

Kinematic measures: *t_C_* ^−1^ (inverted contact duration), *t_S_* ^−1^ (inverted swing duration), *s_S_* (swing length), and *s_T_* (stride length). Medians as well as 25% and 75% percentiles (in parentheses) are given. Resultant linear functions relate the parameters to running speed. Significance numbers (sig) refer to comparisons with significant differences between legs 1 to 4 (from anterior to posterior) on the 5% level, i.e. if 3 and 4 occur in the significance column of the 1^st^ legs, then the differences of the frontal legs to the 3^rd^ and 4^th^ legs are significant, but not to the 2^nd^ legs. For *s_T_* the transitional speeds *v_×_* were 0.28 ms^−1^ in the first, 0.27 ms^−1^ in the second, 0.39 ms^−1^ in the third, and 0.39 ms^−1^ in the hind legs with much lower increases in the stride lengths of the third and fourth legs than in the anterior leg pairs. In *t_S_* ^−1^ the transitions occur at 0.28 ms^−1^ in the first, 0.25 ms^−1^ in the second, 0.2 ms^−1^ in the third and at 0.28 ms^−1^ in the hind legs.

**Table 2:** Comparisons of mean values of kinematic parameters of the walking legs.

	1st legs (median; Q25/75)	sig	2nd legs (median; Q25/75)	sig	3rd legs (median; Q25/75)	sig	4th legs (median; Q25/75)	sig
*f_T_* [s^−1^] (*v*>0.5 ms^−1^)	6.3 (5.7/6.7)	34	7.2 (5.1/7.7)	34	8.6 (7.9/9.1)	12	8.3 (7.7/9.0)	12
*s_C_* [mm]	33.2 (28.4/37.3)	234	30.1 (22.1/33.4)	14	30.0 (22.1/33.4)	14	24.0 (20.4/26.9)	123
*s_T_* [mm] (*v*<0.33 ms^−1^)	54.2 (35.2/70.1)	-	49.2 (35.1/54.8)	-	46.2 (37.6/52.8)	-	47.5 (37.3/52.7)	-
*s_T_* [mm] (*v*>0.5 ms^−1^)	91.7 (86.6/96.9)	34	84.2 (72.3/104.1)	34	62.6 (58.0/70.2)	12	64.9 (58.9/68.8)	12
AEP [mm]	50.5 (46.2/52.5)	234	42.6 (37.5/46.9)	134	4.9 (−0.9/9.6)	124	−22.0 (−25.7/−18.5)	123
PEP [mm]	16.3 (13.2/19.7)	234	13.0 (7.2/15.6)	134	−23.9 (−27.1/−20.4)	124	−46.1 (−47.6/−44.1)	123
lateral distance [mm]	13.9 (18.6/8.7)	234	29.5 (34.4/24.2)	134	34.5 (36.4/30.8)	124	18.5 (20.9/16.4)	123

Comparisons of means between stride frequencies at high speeds (*f_T_*), contact lengths (*s_C_*), stride length (*s_T_*) at high and low speeds, anterior (AEP) and posterior (PEP) extreme positions, as well as lateral distances of the tarsal contact points of the four pairs of walking legs (1 to 4, counted from anterior) to the body axis. Significance numbers (sig) refer to comparisons between the legs with significant differences on the 5% level, i.e. if 3 and 4 occur in the significance column of the 1^st^ legs, then the differences to the 3^rd^ and 4^th^ legs are significant, but not the difference to the 2^nd^ legs.

**Figure 6:**
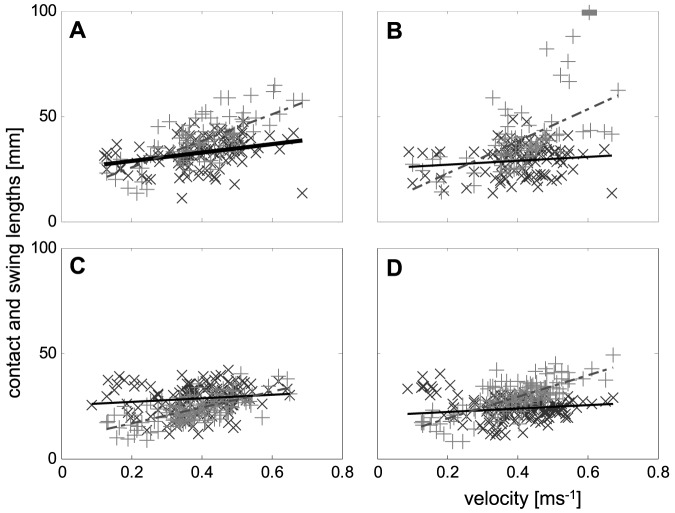
Velocity dependencies of the contact lengths (*s_C_*, black ‘×’ symbols) and swing length (*s_S_*, grey ‘+’ symbols) of all leg pairs. **a**) Front legs, **b**) second legs, **c**) third legs, and **d**) hind legs. The linear regressions of the swing lengths (grey dash-dotted) and of the contact lengths (black solid) are depicted as bold lines.

Contact as well as swing lengths (*s_C_*, *s_S_*) scaled linearly with speed (Figure 6). The contact lengths were almost constant throughout the whole speed-range. That of the front legs was significantly larger and that of the hind legs was significantly smaller than that of all other legs (Table 2). The swing lengths increased with running speed. Here the slope of the 3^rd^ legs was significantly smaller than of all other legs (Table 1). The intercepts with the abscissa showed no differences. In contrast to *s_C_* and *s_S_*, the stride lengths (*s_T_*) of all legs showed bilinear velocity dependencies. They were constant at low running speeds and sloped linearly at higher speeds (Tables 1, 2). While the means at low speeds and the intercepts with the abscissa at higher speeds displayed no differences among the leg pairs, at higher speeds the slope at the front legs was significantly higher than that of the hind leg pairs, resulting in significantly higher *s_T_* of the front legs at high speeds (Table 2).

### Phase relations between the legs' footfalls

Phase relations were used as a measure of the temporal coordination of legs. Ipsilateral and contralateral phase relations were determined. As expected from an alternating locomotion model [44], the contralateral phase relations were all close to 0.5. In contrast to the idea of alternating sets of legs, the phase relation in leg pair 2 showed no clear unimodal distribution. Their phase relations were rather equally distributed around the trigonometric circumference. Consequently, also the coupling was very low (Table 3). All other contralateral phase relations showed unimodal distributions. The leg pairs 1 and 4 showed the strongest contralateral couplings. The analysis of the ipsilateral phase relations revealed a relatively good coupling of 0.54 between the frontal leg pairs as well as a rather high coupling of 0.88 of the hind leg pairs. Phase relations between both groups revealed that the coupling was mostly lower or were even equally distributed (Table 3).

**Table 3:** Phase relations 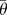, circular standard deviations 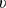, confidence intervals and strength of coupling 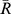 between ipsilateral and contralateral walking legs.

compared legs	mean phase 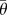	circular standard deviation 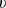	confidence interval	strength of coupling 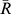	n	Kuiper's test of uniformity
1st legs in 2nd legs	0.50	0.15	0.42/0.57	0.54	98	
1st legs in 3rd legs	0.48	0.22	0.40/0.55	0.09	123	uniform
1st legs in 4th legs	0.84	0.22	0.71/0.98	0.08	120	uniform
2nd legs in 1st legs	0.48	0.15	0.41/0.55	0.54	101	
2nd legs in 3rd legs	0.49	0.18	0.41/0.57	0.36	124	
2nd legs in 4th legs	0.02	0.20	0.02/0.03	0.21	112	
3rd legs in 1st legs	0.24	0.21	0.20/0.28	0.11	119	uniform
3rd legs in 2nd legs	0.51	0.16	0.43/0.60	0.51	134	
3rd legs in 4th legs	0.54	0.08	0.43/0.65	0.88	189	
4th legs in 1st legs	0.39	0.22	0.33/0.45	0.07	117	uniform
4th legs in 2nd legs	1.00	0.17	0.85/1.15	0.42	103	
4th legs in 3rd legs	0.43	0.08	0.35/0.52	0.88	180	
1st legs	0.50	0.10	0.41/0.59	0.81	143	
2nd legs	0.41	0.22	0.36/0.47	0.09	76	uniform
3rd legs	0.49	0.13	0.40/0.58	0.69	158	
4th legs	0.48	0.06	0.41/0.55	0.92	91	

Several phase relations show uniform distributions (Kuiper's test), they therefore lack significant distributional maxima. Circular means, circular standard deviations, and confidence intervals calculated in radians were transformed into fractions of the stride period.

### Translational and rotational DOFs of the body

**Figure 7:**
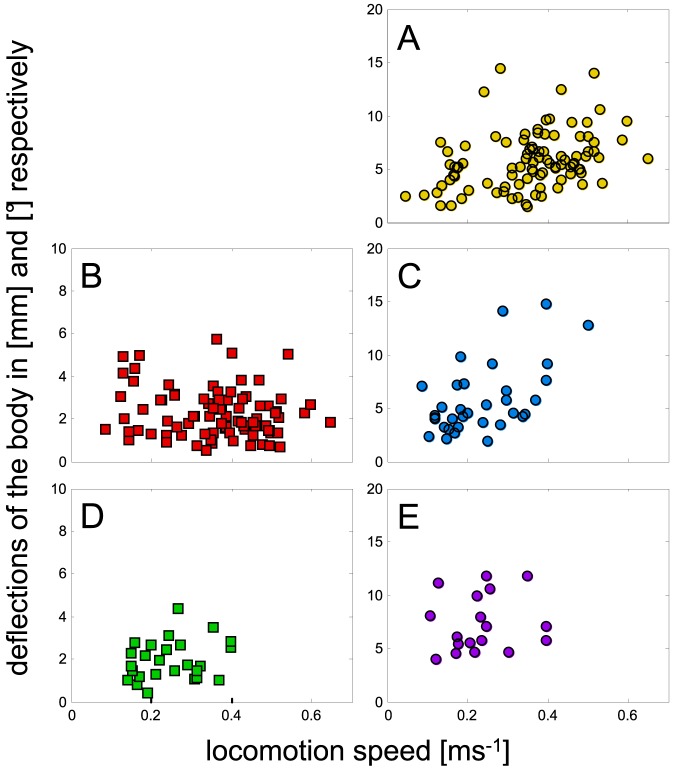
Velocity dependent changes of the DOF's amplitudes. **a**) Yaw, **b**) lateral excursions, **c**) pitch, **d**) vertical excursions, **e**) roll.

In order to analyse the movements of the COM and the body, the COM's translational DOFs and the rotational DOFs of two body axes were analysed. Except for the amplitudes of anterior-posterior fluctuations, the amplitudes and oscillation frequencies are given for all measurements. During running the anterior-posterior position of the COM is dominated by the average forward speed rather than fluctuations about it. Thus, no amplitudes could be extracted for this DOF. All other amplitudes were relatively low. On average, across all DOFs, translational fluctuations cover a range of about 2 mm and rotational fluctuations were about 5°. The median value of lateral displacements was about 2.1 mm (quartiles: 1.4 mm/2.9 mm) and that of the vertical excursions was 1.7 mm (1.2 mm/2.7 mm). The median yaw value was 5.4° (4.0°/7.1°), that of pitch around the transversal axis was 4.6° (3.7°/7.2°), and the median roll value was 6.6° (5.4°/9.9°). Only the excursion values of yaw and pitch showed some statistically significant velocity dependent slopes (Figure 7). While lateral (3.2 s^−1^ (2.8 s^−1^/3.9 s^−1^)) and vertical (3.6 s^−1^ (3.0 s^−1^/4.8 s^−1^)) frequencies showed values close to the mean stride frequency (3.5 s^−1^ (2.4 s^−1^/5.1 s^−1^)) at low speeds (below about 0.27 ms^−1^), there was no further increase at speeds beyond the transitional velocity. The anterior-posterior frequency (about 4.1 s^−1^) showed no velocity dependent slope at all. At low speeds the rotational frequencies also showed values close to that of the stride frequencies of the legs (yaw: 3.2 s^−1^ (2.4 s^−1^/3.7 s^−1^); pitch: 2.9 s^−1^ (2.5 s^−1^/6.1 s^−1^); roll: 4.6 s^−1^ (3.4 s^−1^/6.8 s^−1^)), and at higher speeds none of them showed a significant dependency with regard to running speed (yaw: 7.0 s^−1^ (5.6 s^−1^/8.0 s^−1^); pitch: 6.4 s^−1^ (5.0 s^−1^/10.1 s^−1^); roll: 6.8 s^−1^ (6.1 s^−1^/9.0 s^−1^)).

At high speeds above 0.27 ms^−1^, the values of the translational frequencies remained significantly below the stride frequencies, while the rotational frequencies showed no significant differences to the mean stride frequency. Here, the median values of the translational DOFs (cp. Figures 6a, c, e) did not differ, and those of the rotational DOFs (Figures 6b, d, f) were also not significantly different among each other.

## Discussion

The frequency relations determined in this study clearly differ from those frequency relations expected from model predictions. Thus, the locomotion dynamics of *C. salei* and supposedly of many other large vagrant spiders must also deviate from these model dynamics, resulting in two major consequences: Elastic structures potentially suitable for temporary storage of movement energy, admittedly not yet verified, could not be employed at all with lacking alignment of vertical oscillations of the COM and stride frequencies of the legs. Furthermore, the inherent stabilising mechanisms of spring-mass type locomotor systems also cannot come into effect. How large spiders are able to generate efficient and safe movements in spite of these constraints will be analysed in the following.

### Considerations of running dynamics

**Figure 8:**
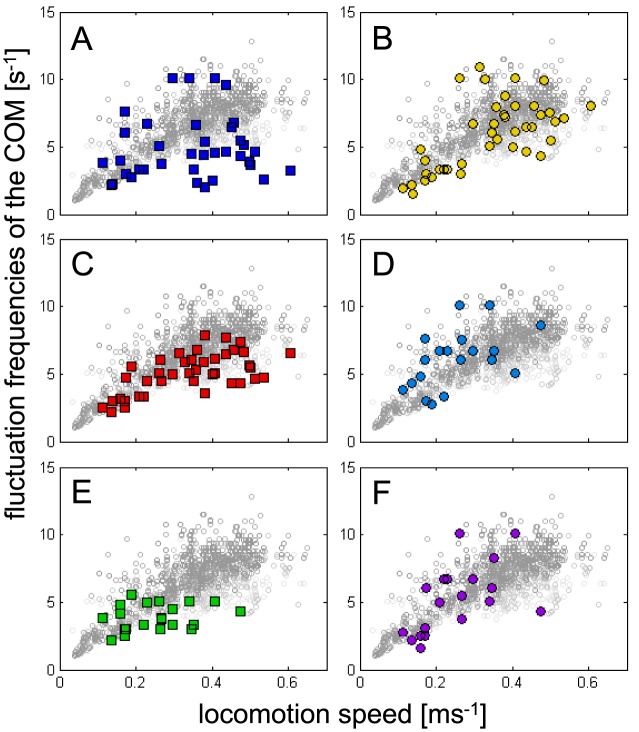
Velocity dependent frequency changes of the COM's translational (a, c, e), and rotational (b, d, f) DOFs of the body. For comparability, all frequencies are shown in front of the stride frequencies of the leg pairs 1 to 4 (small circles in light grey). **a**) anterior-posterior fluctuations, **c**) lateral fluctuations, **e**) vertical fluctuations, **b**) frequency changes of yaw, **d**) pitch, **f**) roll. Particularly at high running speeds fluctuation frequencies of the body's DOFs are significantly lower (translational DOFs) or similar (rotational DOFs) to the stride frequencies of the legs. These characteristics do not comply with the predictions made by spring-mass or inverted pendulum models.

In contrast to observations by Spagna et al. [15], in small agelenid spiders, aerial phases, i.e. instances with no feet on the ground, could not be observed in continuously running *C. salei*. Large spiders seem more likely to have adapted to an inverted pendulum type of locomotion. Kinetic and potential energy of the COM fluctuates out of phase and can, in principle, blend into each other [30], [41]. Nevertheless, as a consequence of low vertical oscillations this energy recovery seems to be principally low. Brüssel [41], for example, determined an energy transfer between kinetic and potential energy of only 8.4% for slow locomotion in *C. salei* [32], [41]. Biancardi established this value for the tarantula *Grammostola mollicoma* as being about 12% and largely independent of speed [30]. However, vertical and anterior-posterior oscillation frequencies had to attain values of twice the stride frequencies in the case of inverted pendulum dynamics. As no such high values could be ascertained (cp. Figure 8), the proposed dynamics, in general, do not apply here.

The strict inverted pendulum model does not cover aerial phases and ballistic behaviour of the COM. However, in running sequences of arthropods aerial phases can only occur if duty factors reach values below 0.5 and leg coordination is close to an alternating pattern. In insects and crustaceans, metachronal leg coordination seems to be the basis of all locomotion patterns, even if it is indiscernible in fast running species using alternating stepping patterns [44], [45]. Regarding energy-efficiency, alternating stepping pattern with aerial phases only make sense if considerable proportions of the movement energy can be stored in elastic elements of the skeleton. Fast running cockroaches, for example, temporarily store kinetic energy in the hip joint of their rear legs [6]. In harvestmen, despite being arachnids moving hexapedally, energy storage seems to rely on elastic properties of their distal legs [46]. To make efficient use of the elastic structures, the legs of a tripod must be tightly coupled and the storing springs have to be loaded and unloaded in accordance with the natural frequency of the spring-mass system. Spring-mass dynamics can prove advantageous since they cause self-stability of locomotor systems in the horizontal as well as in the sagittal plane [19], [25], even if being energetically costly. In this case the fluctuations of kinetic and potential energy would not rely on passive structures, but are rather provided by muscles, which generate and dissipate motion energy. High dynamic stability with low control effort, in turn, may be almost inevitable for fast running on rough terrain [25], [47]. To illustrate this, the lateral leg spring model for the description of lateral fluctuations in running cockroaches is a good example. Although the passive two-legged model perfectly describes global cockroach kinematics and even entails dynamic stability against lateral disturbances [48], [49], the legs of real cockroaches show only little lateral elasticity. The oscillations of cockroaches, from one side to the other, around their running path, results rather from the inherent imbalance of their tripodal sets of synchronously active legs.

### Comparing measured and assumed frequency relations of the body's DOFs

Spring-mass like dynamics, whether based on passive springs or not, imply specific relations between the fluctuations of the COM and the cycle rates of the involved legs. This has already been recognised for cockroaches [21]–[23]. It has been shown that lateral oscillations, yaw and roll fluctuate with the same frequency as the tripods, i.e. at stride frequency, whereas anterior-posterior and vertical oscillations as well as pitch fluctuate with twice the stride frequency. The frequency relations observed in *C. salei* deviate significantly from this pattern (Figure 8). Spring-mass like dynamics are not expected during slow locomotion, here also fast runners like cockroaches revert to metachronal coordination [50]. In *C. salei* all DOFs of the body adopt similar frequencies and follow the velocity dependent increase of the stride frequencies. This also contrasts against the requirements of the inverted pendulum model (see introduction). However, even during higher velocities, the frequencies of the body's DOFs do not behave in accordance with the ratios expected from the model predictions. Whereas stride frequencies grow throughout the whole speed-range, the frequencies of the COM's translational DOFs saturate at values of about 5 s^−1^. The rotational DOFs of the body roughly adopt stride frequencies. Hence, in accordance with the low coupling ratios between the frontal and rear leg pairs, as well as between the contralateral 2^nd^ legs, the locomotor system of *C. salei* apparently does not adopt spring-mass dynamics during fast running. Nevertheless, even if considering gallop dynamics, the measured frequency relations do not match. Gallop, being an asymmetrical gait [51], requires a frequency relation of 1∶1 between vertical oscillations of the COM and stride frequency [52]. Thus, the locomotor system as a whole should not provide a physical basis for self-stability.

### Considerations of running stability

Although slower than the fastest running insects, maximum sprint speeds of vagrant spiders are considerable. These fast motions as well as their ability for fast accelerations [16] enable them to easily surprise prey and avoid detection by predators. Although both insects and spiders exhibit sprawled posture with distributed mechanical interaction with the ground, the relative mean body height of running *C. salei* is very low, even in contrast to most fast-running insects. As a measure for the relative height, we can use the quotient of body height and the distance to the anterior rim of the supporting polygon spanned by the legs in contact with the ground. This rim, in turn, can be defined by the anterior extreme positions of the 1^st^ and 2^nd^ legs and the connecting line between them. With a mean COM height of about 10 mm and an anterior stability margin at touch-down of about 46 mm (cp. Figure 1), this quotient is 0.21 in *C. salei*. The same quotients calculated for the cockroach *Blaberus discoidalis*, the wood-ant *Formica pratensis* and the desert ant *Cataglyphis fortis* are about 0.5 and even exceed 1 in the slow potato beetle (calculated from values in [39], [53]–[55]). Only the extraordinary slow stick insect *Carausius morosus* shows a similarly small quotient of 0.32 [56]. By proceeding equivalently in the lateral direction and assuming the most lateral contact point as a determining measure, we can assess the maximum lateral stability margin. Here again the quotient was lowest for *C. salei*, approaching 0.29, however those of the insects were never below 0.49 (*F. pratensis*). Thus, the static stability of *C. salei* is significantly higher than that of many insect species during level locomotion. The distinct track widths and mean contact areas of the different leg pairs are results of this extremely sprawled posture. They prevent interferences of the comparably long spider legs, particularly at highest cycling frequencies during fast locomotion (cp. [57] with regard to the fast running centipede *Scutigera coleoptrata*).

For low vertical fluctuations and low body rotations, the quotient of the horizontal inertial force and the weight of the spider body roughly determine the position of the mean point of force application. For *C. salei* mean rotation angles only of about 5° were determined, angles of 15° were not exceeded in any case; not in pitch, yaw, or roll. During continuous locomotion maximum anterio-posterior accelerations of the spiders COM (determined by differentiating positional data twice with respect to time) were about 3.7 ms^−2^ (2.6/4.8 ms^−2^), which is small compared with gravitational acceleration. Therefore, the mean angle of attack can be assessed to be steeper than 70°, while the angle between the line from the anterior stability margin to the COM and the substrate approximately draws an angle of only 20°. Thus, the point of force application was always securely inside the anterior-posterior rims of the supporting polygon. Since lateral accelerations were even smaller, this is also true of the lateral direction. The extremely sprawled legs of spiders seem to provide sufficient static stability to prevent tumbling during fast locomotion and possibly even allow for aerial phases in other species [15].

Although most spiders have relatively long legs, allowing for large stride lengths and consequently relatively low stride frequencies, the stride frequencies of *C. salei* were considerably high in fast running sequences. Thus, the contact durations were also low, and the short time intervals limited potential sensory feedback. In a reflex circuit of sensor hairs on the ventral surface of the coxae, Eckweiler and Seyfarth [58] measured response durations of respective coxa muscles of only 23.2 ms. In reflex loops with lyrifom slit sense organs of the tibiae as the sensory component, the latency was about 39 ms [58], [59]. As these latencies are as long as, or even longer than, contact durations at high running speeds, the corresponding reflexes are supposedly too slow to provide sufficiently fast reactions to disturbances. Furthermore it has been shown that a complete deafferentation of all tibial lyriform slit sense organs did not affect the leg coordination and running ability in *C. salei* at all [60]. Thus, feedback loops seem to be of minor importance during these spiders' level locomotion. However, plurisegmental interneurons were found that might permit reactions of adjacent legs to stimuli acting on proprioceptors of an affected leg [32]. This organisation is probably a kind of regulation of feedback and feedforward control. Leg action might rely on information of a preceding leg, which has already lost ground contact at this time. This way it can probably foster dynamic stability during running.

Most certainly, there are also fast running arthropods where static stability is insufficient and a kind of dynamic stability prevents them from falling. However, dynamic stability is not necessarily a result of spring-mass dynamics. Fast cockroach locomotion for instance is a paradigm for self-stable resistance against lateral disturbances [49]. Nevertheless, by computing a two-legged sagittal spring-mass model resembling running gaits of cockroaches, Srinivasan and Holmes [61] could not find self-stable behaviour. Their model did, however, show small vertical amplitudes and a wandering point of force application in such a way that it was always close to the anterior-posterior position of the COM. Since cockroaches also benefit from having good static stability [23], exploiting additional stability strategies such as self-stability is probably unnecessary to prevent hazardous falls. Here, similarly as in spiders, locomotor regimes providing for dynamic stability in the sagittal plane seem to work on a more local scale. Even if there is no biomechanical basis for self-stable characteristics in the locomotor system of *C. salei*, dynamic stability does not need to rely on mechanical responses such as bouncing. Regardless of whether leg coordination is alternating or rather metachronal, the movement cycles of the legs, repeatedly executing contact and swing phases, allow each single leg to readjust its initial state to potentially changed favourable rheonomic constraints [‘predetermined patterns’ 62], i.e. local feedforward adjustments, if necessary. Thus, the cycling itself has to avert legs to get stuck and may permit stability.

### Causes for low leg coordination

*C. salei* realizes high running speeds by increasing both stride frequency and stride length. Stride lengths increase by lengthening the distances that the COM travels during leg swing while contact length is kept almost constant (Figure 6). At low speeds the stride frequencies of the different legs showed similar velocity dependent slopes (Figure 3), which indicate continuously alternating sets of legs or at least coordinated metachronous sequences of leg movements (cp. Figure 5). At high speeds, in contrast, the stride frequencies of the frontal and rear leg pairs deviate significantly. Here the stride frequencies of the 3^rd^ and 4^th^ legs are about 1.5 s^−1^ higher than in the frontal leg pairs (Table 2, Figure 3). Hence, there is no symmetrical stepping pattern with two alternating sets of diagonally adjacent legs. At first glance, the legs act in two loosely coupled groups such that the hind legs cycle faster in an almost fixed phase relation, whereas the ipsilateral coupling of the slower cycling 1^st^ and 2^nd^ legs is lower. This relationship seems to mirror the different relative lengths and functions of the walking legs, and clearly reflects the significantly longer stride length of the frontal leg pairs (Table 2). Similar results were also found by Ferdinand [63] for the much smaller crab spiders (Thomisidae). These spiders are characterised by considerably longer frontal leg pairs. The 1^st^ and 2^nd^ legs are up to twice as long as 3^rd^ and 4^th^ legs. In *C. salei* the frontal leg pairs are also significantly longer than the rear leg pairs [16] and contribute to the animal's propulsion by means of muscularly driven leg flexions, whereas the 3^rd^ legs are considerably shorter and the 4^th^ legs must extend during stance. As the hydraulic leg extension mechanism of small spiders provides sufficiently strong leg extension allowing for considerable thrust [64], different leg lengths are probably the major reason for the irregular stepping patterns in crab spiders. Analogous findings were also made in grasshoppers where the much longer rear legs show significantly lower cycling rates [65]. The higher lateral variability of the frontal leg pairs in *C. salei* is probably a result of their greater lengths, as it allows for variation in ground contact without affecting the COM dynamics. However, it might also indicate a dominant role of the frontal legs in determining and stabilising the course of the spiders' path.

### Effects of size and the efficiency of hydraulic leg extension

However, in contrast to small species, recent findings in jumps of the large species *Ancylometes concolor* proved that the hydraulic mechanism is insufficient for power generation in large spiders [7]. Thus, also during fast continuous locomotion, ground reaction forces of the rear leg pairs should be generated mainly by flexor muscles in the functional hips. Due to relatively unfavourable outer lever arms, these muscles must generate high joint torques. As hip muscles are only short [66] they probably cannot provide sufficient tension throughout the whole possible range of stride lengths of the rear legs, therefore allowing effective propulsion only in the central part and shortening the available contact length (cp. Table 2). Finally, the hydraulic mechanism may even hamper the protraction of the hind legs. Since high pressures [67], [68] are necessary during fast locomotion for fast extensions of protracting frontal leg pairs, the pressure may obstruct efficient hind leg flexion during swing [7]. Thus, the contact length and consequently the contact durations should be limited and may be the reason for a higher cycle rate.

**Figure 9:**
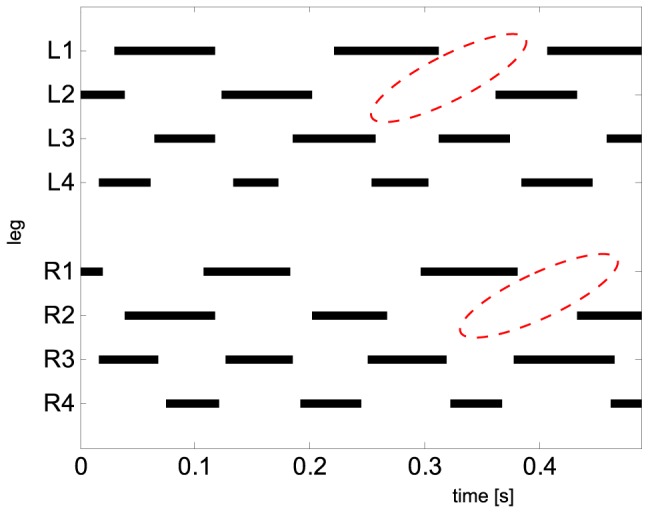
Gait diagram showing the stepping pattern of a typical fast running sequence. The animal ran at approximately 0.5 ms^−1^. Though seemingly a typical metachronal wave pattern with largely synchronous tetrapods of diagonally adjacent legs, on average the frontal legs show longer contact, swing and consequently stride durations than the third and fourth legs (cp. Figures 2, 3 and Table 1). At about 0.25 to 0.3 s the left second leg skips a contact phase and subsequently also the left front leg omits ground contact. The same scenario happens on the right side between about 0.33 s and 0.45 s. (each indicated by dashed red ellipses). The frontal legs fit in back into the normal pattern of the temporal wave when the next one arrives from the posterior leg pairs. During such omissions, the second legs often showed a lowering of the distal leg tips that typically precedes ground contact, and on failing they continue directly with the next swing.

From a neuronal point of view, leg cycling in arthropods is often considered as being only loosely coupled [e.g. 45], [69]. The typical coupling found in different gait patterns, like the metachronal wave and alternating sets of diagonally adjacent legs, is to some extent a result of mechanical requirements and constraints [45], [70]. No matter which mechanism (different leg lengths, or insufficiency of the hydraulic mechanism, or muscle architecture) causes the shorter contact length of the rear leg pairs in *C. salei*, the activities of the 4^th^ and 3^rd^ legs should be coordinated as they have to support the heavy opisthosoma. Avoidance of interferences and mechanical entrainment via the substrate with similar cycle frequencies surely contribute to the tight coupling of these legs. The 2^nd^ legs are longer and they contribute to the animal's propulsion by means of muscular leg flexion [71]. By this, they have a wider range and can use the entire range for the effective generation of propulsive ground reaction forces. By exploiting their full contact length, the cycle frequency and, therefore, the rate-dependent energy consumption can be reduced. To make use of this capacity, while still having synchronous ground contact with their diagonally adjacent rear legs, depending on running speed, the 2^nd^ legs occasionally have to skip contact phases (cp. Figure 9), however, slight downward movements of the 2^nd^ legs were still visible, but they did not lead to ground contact. As the occurrence of these omissions is quite irregular but not rare, the coupling strengths between frontal and rear leg pairs are mostly low. Since omissions occur at different instants of time in the right and left legs of the 2^nd^ leg pair, the contralateral coupling of these legs showed uniform phase distributions and extraordinary low coupling strength. Similar to the temporal relations of the rear leg pairs, the front legs also have to avoid interferences with the 2^nd^ legs. Consequently, they showed considerable coupling (Table 3). The 1^st^ legs skip ground contact whenever the ipsilateral 2^nd^ leg has already skipped. Only the foregoing contact phase is somewhat prolonged and the next starts a bit earlier, so temporal gaps are compensated and contralateral coupling can achieve much higher values than in the 2^nd^ legs.

Similar irregular leg behaviour has been detected by Shultz [14] for the 1^st^ legs of the fishing spider *Dolomedes triton* and by Ward and Humphreys [72] for the wolf spider *Lycosa tarantula*. Both species are relatively large. Irregular stepping patterns were also found during fast locomotion in theraphosid spiders [30]. As leg lengths show a regular distribution among the leg pairs (cp. also [73], [74]), distinct length differences seem to be insufficient to explain these findings. These so-called tarantulas or bird spiders are mostly very large. Therefore it is likely that the hydraulic mechanism, which is only of limited use for the generation of thrust in the hind legs of much smaller *A. concolor* [7], contributes only marginally to the power generation of these big animals. As irregular stepping patterns occurred only during fast locomotion in tarantulas, this behaviour seems to support the hydraulic insufficiency hypotheses.

### Speed dependent changes

At low speeds the distances from touch-down to take-off and from take-off to touch-down of a certain leg's tarsus are equal. The legs are swung forward while the contralateral legs were retracted during their contact phase. However, due to relatively shorter swing durations in comparison to contact durations (cp. Figure 2) and overlapping contact phases, the distance travelled by the COM during the swing phase was smaller, but increased with speed (Figure 6). In contrast, the distance travelled by the COM during contact is almost constant for all speeds. Hence, at a certain speed the swing length exceeds the contact length. At higher speeds, the contact phases of mostly alternating contralateral legs no longer overlap. In the 1^st^, 2^nd^ and 4^th^ legs this transition occurs at relatively low speeds of about 0.27 m/s. Mainly because of the low slope of the swing length, the transition occurs considerably later in the 3^rd^ legs at about 0.53 m/s. Consequently, the contact phases of the 3^rd^ legs overlap even during fast running. Therefore, they seem to bear a major part of the vertical ground reaction forces, quite similar to standing [41] or running *Ancylometes concolor* [75], even if legs are not organized in alternating sets. If the legs were organised in alternating quadruped sets, then instances where only a single 3^rd^ leg is on the ground should occur regularly at speeds above 0.27 m/s, since the contralateral legs of this leg pair alternate consistently. Indeed such strict coordination patterns are rare in spiders [29], [63], [76] and could also not be determined for *C. salei* in this study (see results). So, even in small spiders with a roughly alternating gait pattern, Spagna et al. found synchrony factors, i.e. a normalized fraction of stride overlap between legs in the same tetrapod, of only 0.3 [15]. In relation to leg lengths, vertical oscillations of the COM were always low. Accordingly and in contrast to small agelenid spiders, aerial phases could not be observed in *C. salei*.

The lower slopes of the inverted swing durations (*t_S_* ^−1^) at higher speeds, which are particularly evident in the 1^st^ and 2^nd^ legs, are surely a consequence of changing ratios between contact and swing length and the resulting decrease in double support of synchronously active legs. At high speeds the slopes of inverted swing durations are significantly lower in the frontal leg pairs (Table 1). The almost constant values (Figure 2) seemingly mirror the longer stride lengths and the overall lower cycling frequency in comparison to the rear legs. However, transitions also occurred in the relationships of contact and swing durations. At higher velocities, when velocity dependent slopes of the inverted swing duration are low, the contact durations become shorter than the swing durations. Similar to the contact and swing lengths relationship, the intersection points of contact and swing durations occurred in the 1^st^, 2^nd^ and 4^th^ legs at lower speeds and relatively late, i.e. at a higher running speed, in the 3^rd^ legs (Figure 2). In accordance with the changing ratios, in the 1^st^, 2^nd^ and 4^th^ legs at high speeds the mean duty factors sank below 0.5 (Figures 2, 4). In the 3^rd^ legs, duty factors only scarcely attained values below 0.5, which again supports the vital role of the 3^rd^ legs in the generation of vertical ground reaction forces.

Most of these nonlinear changes in the velocity dependencies of several parameters (cp. Figures 2–5 and Table 1) occur in small speed ranges. They indicate distinct transitions (cp. [77]) in spider locomotion, which are visible in the kinematics of the walking legs but do not, or only marginally, find an expression in the dynamics of the body. Thus, in *C. salei*, the relatively small fluctuations of the COM at all running speeds occur within a limited frequency range. A proximate cause can be proposed in the weak leg coordination.

As ground contacts do not occur in a synchronised fashion, the momentum of the COM might be expected to change at frequencies higher than the stride frequency of a single leg. Nevertheless, due to the variously overlapping contact phases of the walking legs, the lower frequencies caused by the interferences of asynchronously applied ground forces seem to dominate the locomotion dynamics. A more detailed investigation of ground reaction forces in *C. salei*, here, could elucidate the roles of the single legs in these complex interactions.

### Physiological considerations

It has been found that the mass-specific cost of transport in spiders is in the same range as in other similar sized legged animals [78]. However, during fast running, spiders can accumulate substantial oxygen debts, which may reduce the apparent cost of transport considerably [79]. Therefore, the running time decreases regularly with increasing speed, indicating increasing anaerobic contributions [78]. The lack of mitochondria in spider leg muscles [80] surely contributes to this situation. Furthermore, oxygen supply of the prosoma and the legs rely on gas exchange at the book lungs in the opisthosoma and the transport of the oxygenated hemolymph into the anterior body parts. During vigorous movements, such as jumping and fast running, the internal hemolymph pressure in the prosoma reaches very high values [67], [68]. Consequently, the hemolymph flow from the opisthosoma to the prosoma and therefore the oxygen supply of the leg muscles is also largely impeded. High-energy phosphates like arginine phosphate, which are usually depleted in the first seconds of vigorous activities [81], therefore, cannot be restored and energy supply of the muscles is consequently low afterwards. As the capability for sufficiently sustained escapes is still crucial, energy saving mechanisms are likely to be particularly important for spiders. Thus, as a kind of ultimate cause, the weakly coupled legs may also bear an energetically advantageous temporal distribution of the ground contacts (see below).

Due to the larger sum of the distances between the joints and the leg axes, the compliance of the roughly three-segmented bow-shaped legs of spiders is increased as compared to zigzag shaped [82] or two-segmented legs [83]. Furthermore, no passive elastic structures have been found in spider legs so far [8], [84]. Bouncing gaits seem to be particularly costly with such compliant but inelastic legs [85]. The typical rhythmic oscillations of kinetic and potential energy have to be completely generated by sequentially force generating and dissipating muscles. Thus, low fluctuation amplitudes and as low as possible frequencies should be more energy-efficient. Both objectives are achieved best by temporally distributed foot contact providing almost constant overall vertical ground reaction forces.

Relatively slow fluctuations of the COM and higher cycling frequencies of the legs may indicate another strategy to reduce metabolic effort. With shallow trajectories of the COM, i.e. low vertical amplitudes, Ruina et al. [52] showed for completely inelastic locomotor systems moving kinematically similar to spring-mass structures, that the change of movement energy and therefore the metabolic costs depend on the number of impulses, which cause the directional change from downwards to upwards. In the case that a certain angular change of the COM trajectory is the result of two impulses instead of one, then only half of the energy is dissipated. Spreading total impulse into *n* sub-collisions will reduce the energy expenditure by the factor *n* [52]. As vertical amplitudes and pitch angles are low in *C. salei* and neither clearly alternating sets of legs with tight coupling nor the typical frequency relations of spring-mass models were observed, the breakup of tight coupling between the legs of alternating sets of legs may be used by the spiders in order to confine the metabolic effort of fast locomotion.

### Effects of many legged body plans

Even if *C. salei's* leg coordination seems to be quite similar to that of insects, the dynamics of its fast locomotion rather resembles the COM dynamics of fast running centipedes such as *Scutigera coleoptrata*. These animals are also characterised by long walking legs and small fluctuations of the COM [57]. With a body length of 22 mm and contact length of about 33 mm they reach maximum speeds of 0.42 ms^−1^ [86]. Therefore, with increasing leg numbers and elongated walking legs allowing for long contact length, the need for leg coordination seems to result primarily from the necessity to avoid spatiotemporal interferences of these legs. Due to inherently increasing stochastic variations in the coordination of an increasing number of synchronously active legs and the resulting blurred collective ground reaction forces, greater control effort is to be expected for the purposeful generation of supposedly beneficial body fluctuations. Otherwise, just the virtual lack of fluctuations, caused by temporally distributed ground contacts, may provide energy-efficiency (see above). Therefore, in fast running animals with more than six active legs, the synchronisation of two alternating sets of legs should generally play just a minor role.

### Conclusion

For level continuous locomotion in large spiders of the species *C. salei* it was shown that all DOFs of the body show only small oscillations at unexpected low frequencies, particularly at higher speeds. These findings are probably the result of low degrees of coupling between the walking legs as compared to fast running insects. As the established model concepts of fast legged locomotion require distinct frequency relations between body fluctuations and stride frequencies, large spiders obviously use neither spring mass dynamics nor an inverted pendulum mode of locomotion. Consequently, when looking for dynamic similarities, the locomotion of spiders cannot be affiliated to running and walking, i.e., to gaits that are primarily characterised by a high degree of coherent exchange of potential or spring stored energy and kinetic energy of the main body mass. Fast locomotion in spiders is rather a ‘wheel like’ motion [87] and, despite attaining much higher speeds, is dynamically similar to crawling in many slow moving legged arthropods.
